# Underweight as a risk factor for respiratory death in the Whitehall cohort study: exploring reverse causality using a 45-year follow-up

**DOI:** 10.1136/thoraxjnl-2015-207449

**Published:** 2015-08-07

**Authors:** Mika Kivimäki, Martin J Shipley, Joshua A Bell, Eric J Brunner, G David Batty, Archana Singh-Manoux

**Affiliations:** 1 Department of Epidemiology and Public Health, University College London, London, UK; 2 Centre for Cognitive Ageing & Cognitive Epidemiology, University of Edinburgh, Edinburgh, UK; 3 Centre for Research in Epidemiology and Population Health, INSERM, Villejuif, France

**Keywords:** COPD epidemiology

## Abstract

Underweight adults have higher rates of respiratory death than the normal weight but it is unclear whether this association is causal or reflects illness-induced weight loss (reverse causality). Evidence from a 45-year follow-up of underweight participants for respiratory mortality in the Whitehall study (N=18 823; 2139 respiratory deaths) suggests that excess risk among the underweight is attributable to reverse causality. The age-adjusted and smoking-adjusted risk was 1.55-fold (95% CI 1.32 to 1.83) higher among underweight compared with normal weight participants, but attenuated in a stepwise manner to 1.14 (95% CI 0.76 to 1.71) after serial exclusions of deaths during the first 5–35 years of follow-up (P_trend_<0.001).

## Introduction

Underweight individuals appear to have an increased risk of dying from chronic respiratory disease.^1–3^ In the Prospective Cohort Studies Collaboration of 900 000 adults, for example, each 5-unit *decrease* in body mass index from 25 to 15 kg/m^2^ was associated with a 1.7-fold *increase* in respiratory mortality.^1^ This excess risk was evident in men and women and across adult age groups; other studies have reported similar findings.^1–3^

The clinical importance of these findings has been the subject of debate because it is unclear whether the association is causal or a consequence of illness-induced weight loss prior to study baseline (reverse causality). If the first interpretation were true, physicians might encourage their patients to maintain normal weight as a means of preventing respiratory disease.^4^ In the latter scenario, respiratory diseases such as COPD develop over many years and weight loss may precede diagnosis. In addition, some risk factors for respiratory disease, such as smoking, are also associated with weight loss, and may therefore be a further source of reverse causation bias.

The likelihood that death is caused by an existing weight-modifying condition is highest in the earliest stages of follow-up and progressively diminishes over time. Accordingly, if the strength of the underweight–mortality association attenuates when deaths occurring in earlier years of follow-up are removed from the analyses, reverse causation is a likely explanation. In the present study, we examined this issue by comparing the risk of respiratory death among underweight versus normal weight adults over an exceptionally long follow-up period of 45 years.

## Methods

Data are drawn from the original Whitehall cohort study of 19 019 male London-based government employees, aged 40–69 years at study baseline in 1967–1970 (for details, see online supplementary appendix).^5^ Mortality surveillance was available up to 30 September 2012, allowing us to undertake analyses excluding participants who had a respiratory death in the first 5–35 years of follow-up. To examine whether these exclusions cause spurious attenuations, we repeated the analyses focusing on the association between underweight and coronary heart disease (CHD) deaths; if our approach is valid then the association should not be affected because weight loss is less likely to occur in the years prior to CHD.

## Results

**Figure 1:**
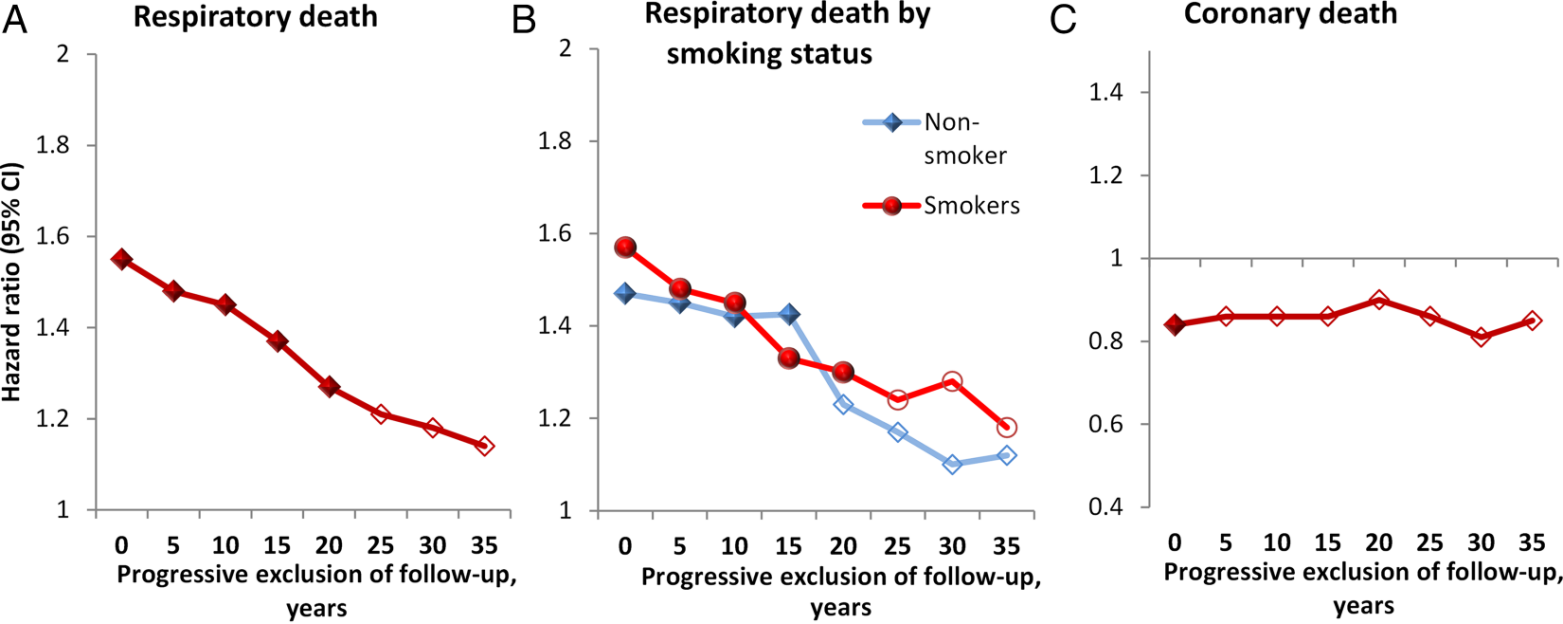
HRs for the association of body mass index (BMI) (underweight vs normal weight) with respiratory (A and B) and coronary heart disease deaths (C) after serial exclusions of the first 5–35 years of follow-up in the total population (A and C) and according to baseline smoking status (B) (filled marks denote statistically significant HR, empty marks statistically non-significant; for data on all BMI categories, see online supplementary appendix eTables 3 and 4).

During 507 648 person-years at risk, 2139 participants died from respiratory disease. Figure 1 shows that the age-adjusted and smoking-adjusted excess risk at baseline was 1.55-fold (95% CI 1.32 to 1.83) higher among underweight compared with normal weight participants (figure 1A). This HR attenuated in a stepwise manner to 1.14 (95% CI 0.76 to 1.71) after serial exclusions of deaths during the first 5–35 years of follow-up (P_trend_<0.001). Results from stratified analyses for current smokers and non-smokers revealed the same pattern of associations (figure 1B). In contrast, analyses of CHD deaths (N=4461) showed 15% lower risk among underweight relative to normal weight participants (HR=0.85, 95% CI 0.71 to 0.99), which remained unchanged after excluding participants who died during the first 35 years of follow-up (figure 1C). Detailed results can be found in the online supplementary appendix.

## Discussion

Exclusions of data are, in effect, subgroup analyses, raising the question whether findings could be an artefact of random variability resulting from reduced sample size or bias due to the exclusions themselves. The consistent stepwise attenuation of the HR for underweight at each additional 5-year exclusion in our data, both in the total population and in smokers and non-smokers, suggests that random variability is an unlikely explanation for our findings. Furthermore, the expected unchanged HRs for the association between underweight and CHD show that exclusions themselves do not bias effect estimates towards the null.

Taken together, evidence from the longest follow-up to date suggests that the observed association between underweight and elevated respiratory mortality is mainly attributable to reverse causation, such that underweight is a consequence and not a cause of respiratory disease or its risk factors. The Prospective Studies Collaboration, the largest study on this issue to date, excluded participants who died within the first 15 years of follow-up^1^ while other studies typically exclude mortality within the first 3 or 5 years. Our findings suggest that these exclusion periods are too short, as the confounded effect of underweight persists much longer.[Table THORAXJNL2015207449TB1]

**Table THORAXJNL2015207449TB1:** Number of participants alive and number of respiratory or coronary heart disease deaths at start of follow-up (0 years excluded) and after exclusion of 15 and 30 years of follow-up

Follow-up excluded (years)	Total population			Non-smokers (smokers)			Total population		
	0	15	30	0	15	30	0	15	30
Underweight									
N (total)	934	704	361	342 (592)	292 (412)	177 (184)	934	704	361
N (deaths)	168	124	53	44 (124)	39 (85)	19 (34)	158	106	36
Normal weight									
N (total)	9397	7774	4436	5012 (4383)	4411 (3361)	2824 (1611)	9397	7774	4436
N (deaths)	1146	1009	545	494 (652)	457 (552)	289 (256)	2017	1442	563

## Supplementary Material

Web supplement
