# Supplementary material for: Underweight as a risk factor for respiratory death in the Whitehall cohort study: exploring reverse causality using a 45-year follow-up
Source: Thorax. 2015 Aug 7;71(1):84–5. doi: 10.1136/thoraxjnl-2015-207449 (PMC4717419; doi:10.1136/thoraxjnl-2015-207449)
Supplement: Web supplement [file thoraxjnl-2015-207449-s1.pdf]

APPENDIX

Additional data to the research letter "Kivimäki M, Shipley MJ, Bell JA, Brunner EJ, Batty GD, Singh-Manoux A. Underweight as a risk factor for respiratory death in the Whitehall cohort study: Exploring reverse causality using a 45-year follow-up. *Thorax* 2015."

Measurement of body mass index

Height and weight at the baseline examination were measured by a nurse using standard protocol. Body mass index (BMI) was computed as weight(kg)/height^2(m^2) and categorized into: underweight (<20.0kg/m^2) normal weight (20.0–<25.0, reference), overweight (25.0–<30) and obese (≥30.0).

Ascertainment of deaths

A total of 18,863 men (99.2% of participants in baseline survey) were traced for mortality using the National Health Service Central Registry. Causes of deaths were ascertained from death certificates using ICD-8 and ICD-9 codes 460-519 and ICD-10 codes J00-J99 for respiratory disease and ICD-8 and ICD-9 codes 410-414 and ICD-10 codes I20-I25 for CHD.

Statistical analysis

The present analyses are based on 18,823 participants with complete data on BMI, smoking and cause-specific mortality. To compare the association between underweight and normal weight for mortality outcomes, hazard ratios and accompanying 95% confidence intervals (CI) were computed using Cox proportional hazards regression with age during follow-up as the time scale and stratification by birth cohort.

Results

eTable 1. Baseline characteristics of participants

**Characteristic**	**N**	**%**	**Mean (SD)**
Mean age	18823		52.0 (6.7)
Smoking status			
Current cigarette smoker	7840	41.7	
Pipe or cigar smoker only	648	3.4	
Ex-smoker	6865	36.5	
Never smoker	3470	18.4	
Mean BMI			
Underweight (BMI<20)	934	5.0	
Normal weight (BMI 20-24.9)	9397	49.9	
Overweight (BMI 25-29.9)	7691	40.9	
Obese (BMI≥30)	801	4.3	
Mean follow-up, y			26.9 (12.2)
Respiratory death	2139	11.4	
Coronary heart disease death	4461	23.7	

eTable 2. Association of BMI category with respiratory disease deaths after serial exclusions of deaths during the first 5 to 35 years of follow-up

Exclusion	N (total)	N (respiratory death)	Hazard ratio* (95% CI)
No exclusion			
Underweight	934	168	1.55 (1.32, 1.83)
Normal weight	9397	1146	1.0 (Ref)
Overweight	7691	746	0.85 (0.77, 0.93)
Obesity	801	79	1.19 (0.95, 1.50)
First 5-years excluded			
Underweight	880	156	1.48 (1.25, 1.76)
Normal weight	9001	1125	1.0 (Ref)
Overweight	7360	736	0.85 (0.78, 0.94)
Obesity	757	79	*1.23 (0.98, 1.54)*
First 10-years excluded			
Underweight	799	144	1.45 (1.22, 1.72)
Normal weight	8481	1083	1.0 (Ref)
Overweight	6802	704	0.85 (0.77, 0.94)
Obesity	677	76	1.26 (1.00, 1.60)
First 15-years excluded			
Underweight	704	124	1.37 (1.14, 1.65)
Normal weight	7774	1009	1.0 (Ref)
Overweight	649	649	0.85 (0.77, 0.94)
Obesity	72	72	1.35 (1.06, 1.72)
First 20-years excluded			
Underweight	588	101	1.27 (1.04, 1.57)
Normal weight	6846	906	1.0 (Ref)
Overweight	5280	577	0.86 (0.77, 0.95)
Obesity	577	57	1.26 (0.97, 1.65)
First 25-years excluded			
Underweight	473	76	1.21 (0.95, 1.53)
Normal weight	5710	740	1.0 (Ref)
Overweight	4271	479	0.89 (0.80, 1.00)
Obesity	347	46	1.35 (1.00, 1.82)
First 30-years excluded			
Underweight	361	53	1.18 (0.89, 1.57)
Normal weight	4436	545	1.0 (Ref)
Overweight	3091	324	0.87 (0.76, 1.00)
Obesity	224	30	1.31 (0.91, 1.89)
First 35-years excluded			
Underweight	260	26	1.14 (0.76, 1.71)
Normal weight	3170	294	1.0 (Ref)
Overweight	2109	177	0.93 (0.77, 1.13)
Obesity	134	15	1.39 (0.83, 2.34)

*Hazard ratios are adjusted for age and smoking habit (categories: never smoker, ex-smoker, pipe/cigar smoker only, cigarette smoker)

eTable 3. Association of BMI category with coronary heart disease (CHD) deaths after serial exclusions of deaths during the first 5 to 35 years of follow-up

Exclusion	N (total)	N (CHD death)	Hazard ratio* (95% CI)
No exclusion			
Underweight	934	158	0.84 (0.71, 0.99)
Normal weight	9397	2017	1.0 (Ref)
Overweight	7691	2025	1.28 (1.20, 1.36)
Obesity	801	261	1.92 (1.69, 2.19)
First 5-years excluded			
Underweight	880	147	0.86 (0.73, 1.02)
Normal weight	9001	1874	1.0 (Ref)
Overweight	7360	1886	1.29 (1.21, 1.38)
Obesity	757	239	1.96 (1.71, 2.24)
First 10-years excluded			
Underweight	799	126	0.86 (0.72, 1.03)
Normal weight	8481	1665	1.0 (Ref)
Overweight	6802	1654	1.29 (1.21, 1.38)
Obesity	677	203	1.97 (1.71, 2.28)
First 15-years excluded			
Underweight	704	106	0.86 (0.71, 1.05)
Normal weight	7774	1442	1.0 (Ref)
Overweight	649	1389	1.27 (1.18, 1.37)
Obesity	72	165	1.97 (1.68, 2.32)
First 20-years excluded			
Underweight	588	86	0.90 (0.72, 1.12)
Normal weight	6846	1160	1.0 (Ref)
Overweight	5280	1086	1.27 (1.17, 1.38)
Obesity	577	129	2.08 (1.73, 2.50)
First 25-years excluded			
Underweight	473	61	0.86 (0.67, 1.12)
Normal weight	5710	867	1.0 (Ref)
Overweight	4271	784	1.28 (1.16, 1.41)
Obesity	347	85	2.05 (1.64, 2.56)
First 30-years excluded			
Underweight	361	36	0.81 (0.58, 1.14)
Normal weight	4436	563	1.0 (Ref)
Overweight	3091	469	1.24 (1.09, 1.40)
Obesity	224	42	1.78 (1.30, 2.44)
First 35-years excluded			
Underweight	260	18	0.85 (0.52, 1.36)
Normal weight	3170	284	1.0 (Ref)
Overweight	2109	246	1.36 (1.15, 1.62)
Obesity	134	23	2.25 (1.47, 3.45)

*Hazard ratios are adjusted for age and smoking habit (categories: never smoker, ex-smoker, pipe/cigar smoker only, cigarette smoker)
